# The principles of Nurturing Care promote human capital and mitigate adversities from preconception through adolescence

**DOI:** 10.1136/bmjgh-2020-004436

**Published:** 2021-04-19

**Authors:** Maureen M Black, Jere R Behrman, Bernadette Daelmans, Elizabeth L Prado, Linda Richter, Mark Tomlinson, Angela C B Trude, Donald Wertlieb, Alice J Wuermli, Hirokazu Yoshikawa

**Affiliations:** 1 Pediatrics, University of Maryland School of Medicine, Baltimore, Maryland, USA; 2 RTI International, Research Triangle Park, North Carolina, USA; 3 Department of Economics, University of Pennsylvania School of Arts and Sciences, Philadelphia, Pennsylvania, USA; 4 Population Studies Center, University of Pennsylvania School of Arts and Sciences, Philadelphia, Pennsylvania, USA; 5 Maternal, Newborn, Child and Adolescent Health and Ageing, World Health Organization, Geneva, Switzerland; 6 Nutrition, University of California Davis, Davis, California, USA; 7 Centre of Wxcellence in Human Development, University of the Witwatersrand, Johannesburg, Soutn Africa; 8 Global Health, Stellenbosch University, Cape Town, South Africa; 9 School of Nursing and Midwifery, Queens University, Belfast, UK; 10 Eliot-Pearson Department of Child Study and Human Development, Tufts University, Medford, Massachusetts, USA; 11 Global TIES for Children, New York University, New York, New York, USA

**Keywords:** child health

## Abstract

A comprehensive evidence-based framework is needed to guide policies and programmes that enable children and adolescents to accrue the human capital required to meet the Sustainable Development Goals (SDGs). This paper proposes a comprehensive, multisectoral, multilevel life-course conceptualisation of human capital development by building on the Nurturing Care Framework (NCF), originally developed for the foundational period of growth and development through the age 3 years. Nurturing care (NC) comprises stable environments that promote children’s health and nutrition, protect from threats, and provide opportunities for learning and responsive, emotionally supportive and developmentally enriching relationships. NC is fostered by families, communities, services, national policies and beyond. The principles apply across the life course, endorse equity and human rights, and promote long-term human capital. This paper presents an evidence-based argument for the extension of the NCF from preconception through adolescence (0–20 years), organised into six developmental periods: preconception/prenatal, newborn/birth, infancy/toddlerhood, preschool, middle childhood and adolescence. The proposed framework advances human capital within each developmental period by promoting resilience and adaptive developmental trajectories while mitigating negative consequences of adversities.

Attaining the SDGs depends on strengthening human capital formation, extending throughout childhood and adolescence and supported by NC. Embedded in enabling laws, policies and services, the dynamic NCF components can mitigate adversities, enhance resilience and promote the well-being of marginalised groups. The life-course extension of the NCF is strategically positioned to enhance human capital, to attain the SDGs and to ensure that children or adolescents are not left behind in reaching their developmental potential.

**Summary box**

- Children and adolescents benefit from stable environments that promote health and adequate nutrition, protect from threats, and provide opportunities for learning and responsive, emotionally supportive and developmentally enriching relationships.
- The principles of nurturing care (NC) apply from preconception through adolescence and mitigate adversities, enhance resilience, and promote the well-being of marginalised groups, human rights and human capital accrual.
- The foundations of adolescent agency and social responsibility begin with self-regulation during infancy and continue to develop during childhood.
- The life-course extension of the Nurturing Care Framework is strategically positioned to enhance human development, to attain the Sustainable Development Goals, and to ensure that children and adolescents can reach their developmental potential.

## Introduction

Attainment of the Sustainable Development Goals (SDGs) depends on human capital development in current and future generations, to improve health, well-being and productivity.^1 2^ Human capital accrues over the life course, beginning prior to conception and extending throughout childhood, adolescence, and beyond. SDG attainment, buoyed by the substantial progress in child survival, depends on children and adolescents thriving and meeting their developmental potential with benefits that accumulate across the life course and intergenerationally.^3^

A framework provides a ‘big picture’ view of factors related to human capital.^4^ Although lacking the complexities or testable qualities of theories, frameworks help countries meet the SDGs by informing policies and programmes that enable children and adolescents to attain their developmental potential. This paper uses a combination of deductive (top-down/theoretical) and inductive (bottom-up/empirical) approaches to extend the Nurturing Care Framework (NCF) through childhood and adolescence. The bioecological theory of human development^5^ and principles of relational developmental systems^6^ provide the theoretical basis for a life-course framework. Multicountry reviews, series, commissions and authoritative sources, such as Disease Control Priorities, inform the empirical basis of our comprehensive, multisectoral, multilevel life-course conceptualisation of human capital development.

## Nurturing care (NC) through adolescence

NC, an evidence-based multilayered dynamic concept, promotes human capital development from preconception to early childhood through stable environments that promote health and nutrition, provide protection from threats, and ensure opportunities for learning and relationships that are emotionally supportive and responsive.^7 8^ These NC components are all necessary and interdependent, with no single component being sufficient. Building on the concept of developmental cascades and the interdependence among NC components, compromised functioning in one component may ‘spill over’ and disrupt functioning in other components. For instance, undernutrition may interfere with health and learning, and an abusive environment can hinder socioemotional development. Likewise, improved functioning in one component may spill over and enable functioning in other components.

Human development is marked by sensitive periods when environmental conditions and experiences have intensified influence on brain development.^9^ The plasticity associated with sensitive periods provides opportunities to recover from negative consequences of adversities, thereby positively altering developmental paths.^7 8 10^ Consistent with the bioecological theory of human development,^5^ changes in developmental trajectories occur through bidirectional relationships with surrounding environments, extending from family, school, community and beyond. These relational developmental environments can impact biological, physiological and psychological structures and functions within a life-course trajectory that extends throughout childhood and adolescence.

**Figure 1:**
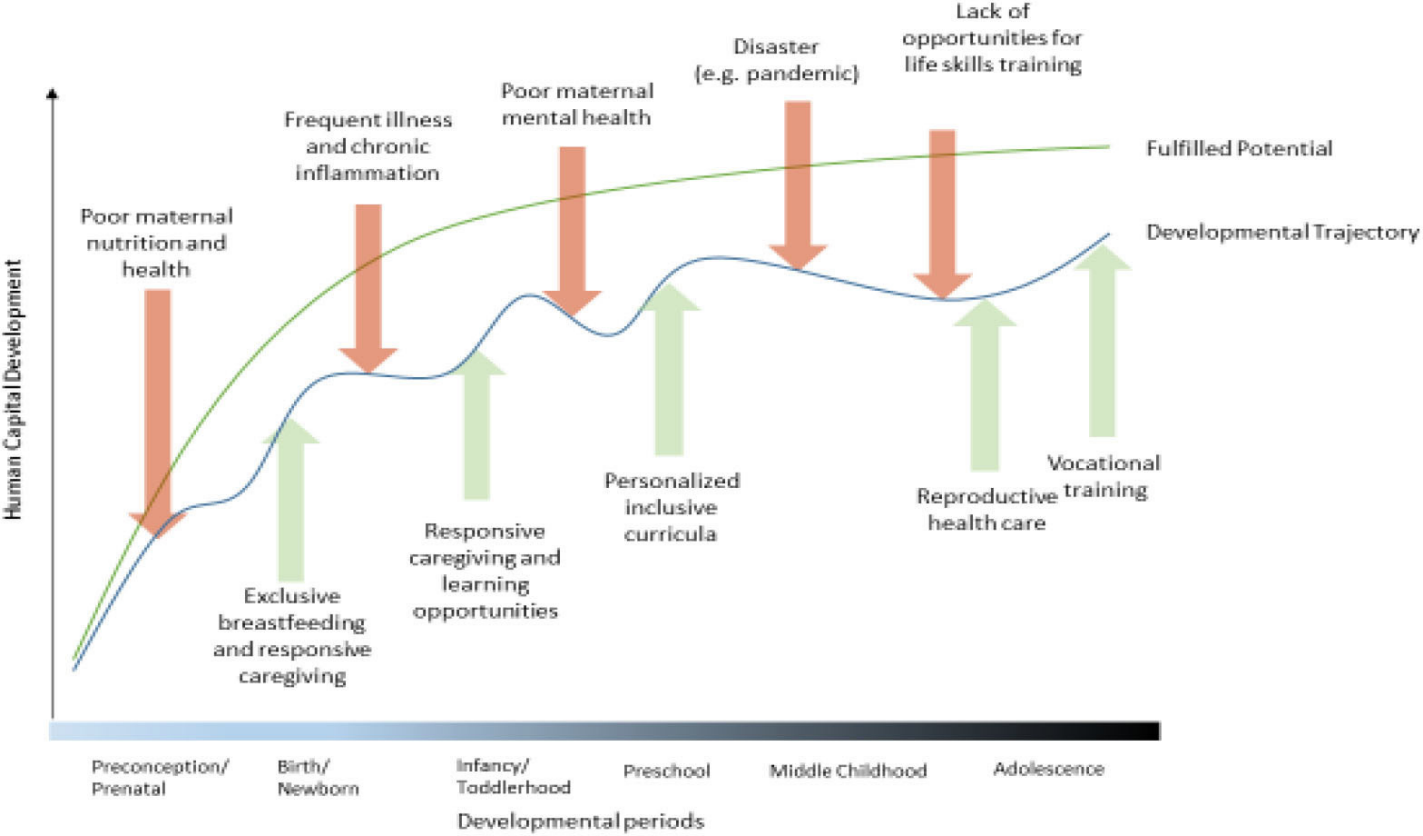
Developmental trajectories from preconception through adolescence.

The NCF incorporates an equity focus by providing a roadmap that transforms child rights into action. Working through multisectoral programmes and policies to alter environmental exposures and strengthen supporting systems, the NCF provides opportunities for positive and compensatory mechanisms.^7^ The processes inherent in NC extend beyond the age of 3 years. Extending the NCF throughout childhood and adolescence with a life-course perspective offers opportunities to support and alter developmental trajectories, to enhance resilience and to mitigate consequences of adversities. Thus, the NCF has the potential to promote human capital development by informing programmes and policies that enable children and adolescents to reach their developmental potential (figure 1).^11–13^

**Figure 2:**
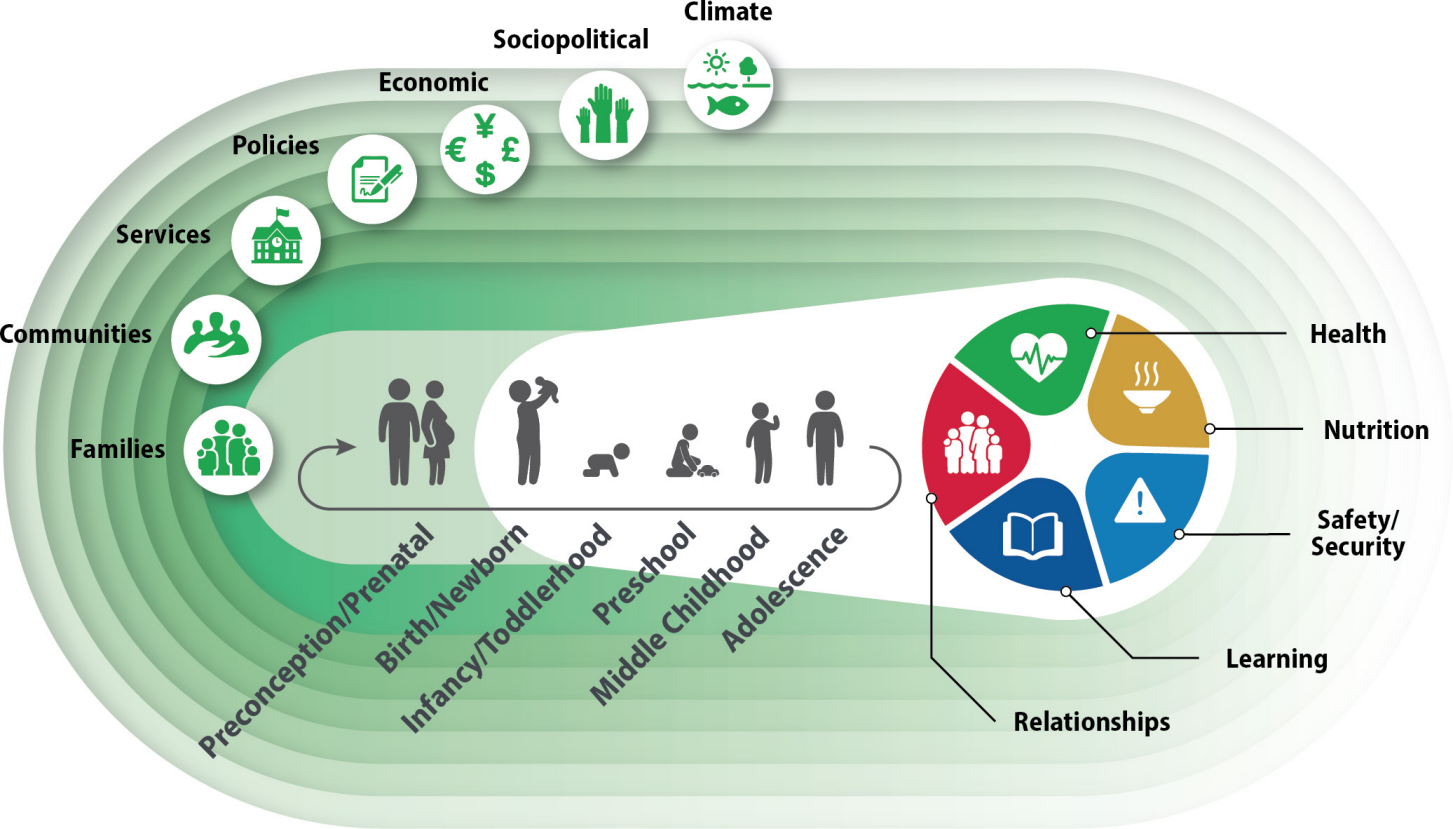
Nurturing Care Framework from preconception through adolescence.

This paper also addresses the WHO–UNICEF–Lancet Commission’s call to invest in the health and well-being of children and adolescents^3^ by reviewing evidence for the application of NC in six developmental periods: preconception/prenatal, newborn/birth, infancy/toddlerhood, preschool, middle childhood and adolescence (figure 2). The following boxes illustrate the application of the NCF to families of children with disabilities (box 1)^14^ and evidence-based principles to mitigate the effects of adversities and promote long-term and intergenerational effects of human capital development (box 2).

Box 1 **Nurturing care (NC) inclusive of children with developmental difficulties, delays and disabilities**

Principles of NC, including the extension from preconception through adolescence, the anchoring in the SDG agenda and the commitment to ‘leave no one behind,’ affirm the important inclusion of children with disabilities. Disability affects at least 93 million children, is both a determinant and a consequence of socioeconomic inequalities, and includes the following research, policy and practice challenges^14^:

- The need for children with developmental delays and behavioural, cognitive, mental and neurological disabilities to have greater access to healthcare, early childhood care and development services, and education.
- The need to have population-level detection, screening, assessment and linkage to evidence-based, multisectoral services to maximise capabilities and social inclusion of children with developmental delays and disabilities.
- The need for educational and care programmes targeted to children and adolescents with delays and disabilities to support their development and the abilities of parents to work to meet financial needs.

Box 2 **Nurturing care (NC) principles to mitigate effects of adversities and promote the long-term and intergenerational effects of human capital**

- The components of NC apply from preconception through adolescence. Nurturing relationships, first within the family and then with peers and community members, are the focus of effective programmes throughout childhood and adolescence.
- To provide the ongoing care, guidance, protection and support that children and adolescents need to achieve human capital, families rely on communities, services, policies and laws to support their physical and mental health, safety, access to services and opportunities to obtain financial stability.
- Multisectoral interventions are needed to build multiple aspects of NC to ensure sustained effects on human capital. Healthcare systems are effective platforms for building learning and parenting interventions in early childhood. Quality childcare services provide learning opportunities and continuity in responsive caregiving for young children. Schools can be leveraged to improve health and nutrition, reduce infectious disease, and address children’s socioemotional development and mental health. Social messages and media are promising avenues to reach youth, parents, and service providers with information and strategies to enhance health, learning and youth-empowering skills.
- Universal policies, including access to free preventive and promotive healthcare, childcare and early childhood education, and quality education from preprimary through secondary, are central to the SDGs. Other critical policies to enhance human capital include bans against child marriage and corporal punishment of children and youth; policies to enhance appropriate nutrition across the life course; and policies to end institutionalisation and promote family care for children.
- Policies that integrate quality education with health and nutrition improve the health and nutrition of children and adolescents. This principle not only improves the ability to learn and become productive adults, but also improves the health of the next generation by creating a long-term cycle of economic growth and progress.^49^
- The reduction of disparities to build human capital requires eliminating inequities and building inclusion. Human rights frameworks require inclusion of children and adolescents with disabilities, addressing the needs of children exposed to conflict, displacement and migration, and reduction/elimination of gender disparities in access to learning and exposure to violence.
- Governmental attention is needed to manage environmental factors that impact the well-being of children and adolescents, including policies related to families, communities and services, along with political, economic and ideological instability, and climate change.

## Developmental periods

### Preconception and prenatal

Preconception health for both partners and women’s health during pregnancy impact fetal development with long-lasting implications for children’s health, growth and development.^15^ A primary threat during the preconception/prenatal period is limited preparation for pregnancy and childbirth associated with unawareness of pregnancy before 6 weeks, thus missing critical windows for intervention.

Exposures to excessive stress, environmental pollutants, nutritional deficiencies, infectious diseases and toxins can cause epigenetic fetal changes and structural and functional abnormalities with consequences that may persist throughout life. For example, folic acid supplementation prior to pregnancy results in a 70% risk reduction in neural tube malformations caused by insufficient folate intake.^16^ Beneficial effects on neonatal and child outcomes have been shown from nutritional supplementation, including multiple micronutrients during preconception and pregnancy.^17^ Evidence from interventions to promote preconception and prenatal health and wellness suggests intergenerational benefits of addressing violence and safety, nutritional supplementation and fortification, cash transfers, healthy diet and exercise, and avoiding teratogenic exposures such as nicotine and secondhand smoke.^18^

Recognition is growing that interventions to promote the next generation’s development must begin prior to conception and must continue through pregnancy.^15^ Promoting future generations’ health and well-being begins with ensuring the health and well-being of adolescents prior to childbearing, thus benefitting two generations, and illustrating the intergenerational cycle of human capital development.^18^

### Birth and newborn

The transition from intra-uterine to extra-uterine life introduces physiological and social changes for newborns and parents, including risks of neonatal death and disabilities.^19^ Infants, born prematurely or small for gestational age, are at increased risk of death in the neonatal period; they are also at increased risk of stunting, developmental delay and disability, and of cardiovascular and other non-communicable diseases later in life.^19^ In addition to the emphasis on health, nutrition and safety during this period, bonding and breastfeeding promote caregiver–child relationships and provide learning opportunities, with lasting consequences for cognitive and socioemotional development.

Breastfeeding provides long-lasting health, nutrition, relationships, learning and protection benefits to infants and mothers. Hormonal responses may reduce maternal stress and enhance mother–infant relationships, potentially enhancing infant cognitive and socioemotional development.^20^ Initiatives, such as the WHO/UNICEF Baby Friendly Hospital Initiative, support skin-to-skin contacts and breastfeeding shortly after birth.^21^ Many countries, including low-income and middle-income countries (LMICs), have adopted breastfeeding promotion policies for working mothers, including paid maternity leave, on-site childcare and facilities for mothers to express milk. These policies benefit children’s health but apply primarily to formal employment, with few benefits for women working in the informal economy.^22^

### Infancy and toddlerhood

Attachment—the emotional relationship between infants and parents—develops during infancy. Through responsive caregiving, infants learn to modulate their behaviours and emotions to accommodate to household routines and to build self-regulatory skills that will eventually enable them to develop future relationships and increase agency during adolescence. Infant development can be disrupted by multiple adversities. Infants born premature or of low birth weight may have difficulty eliciting and responding to responsive caregiving, and parental stress and depression may weaken attachment.^23^ Health, nutrition and safety continue to be important aspects of caregiving during infancy and toddlerhood as, for example, excessive weight gain can lead to lifelong obesity.^24^ Unintentional injuries are a major cause of mortality and disability.^25^ Insufficient quality childcare is a critical concern, particularly with global increases in maternal employment.

Interventions that target responsive caregiving and learning among infants with early risks have benefits that extend to adulthood. Home visiting with psychosocial stimulation among infants and toddlers with stunted growth in Jamaica was positively associated with lower adult crime and higher earnings.^26 27^ Responsive caregiving and early learning opportunities early in life mitigated the impact of early adversities on adolescent IQ in Brazil and South Africa,^13^ and adolescent externalising behaviours in the UK.^28^ Thus, interventions based on the NCF during infancy/toddlerhood, a period of considerable plasticity, can mitigate negative consequences of adversities and improve resilience, developmental trajectories and human capital.

### Preschool ages

Plasticity continues, but lessens, during the preschool years (age 2–5), as early brain development consolidates. With appropriate support, many young children can overcome or lessen the negative effects associated with developmental delays or disabilities.^29^

Health, nutrition and safety remain important during preschool age, including prevention of infectious diseases, undernutrition and obesity, and the detection and management of childhood illnesses. Undernourished or anaemic preschoolers are less likely to explore their environments and interact socially, compared with adequately nourished peers.^30^ Preschoolers who have obesity are at risk of morbidities into adulthood.

By actively engaging in play-based activities, such as climbing, drawing and classifying objects, preschoolers build foundations in motor, cognitive, language and socioemotional skills that enable them to engage in increasingly complex learning, academic and athletic activities.^11^ Preschoolers learn to understand others’ feelings and perspectives and respond appropriately, thus increasing self-regulation and prosocial behaviours. Adversities may threaten human capital by limiting opportunities for social interactions and caregiver engagement, jeopardising children’s health and socioemotional and cognitive development.^11^

One-third of children in LMICs 3 and 4 years of age attend center-based early childhood care and education (ECCE).^31^ These programmes have the potential, when implemented with quality, to enhance young children’s readiness to learn and to improve educational attainment. ECCE programmes also may provide delivery platforms for nutritional programmes, thus addressing hunger and food insecurity and facilitating development of health-promoting habits. Recent reviews of LMIC ECCE programmes showed large positive impacts on learning, particularly among preschoolers from socioeconomically disadvantaged households, as well as beneficial impacts on children’s mental health.^32^

### Middle childhood

Middle childhood, the ‘forgotten years’,^33^ refers to primary school ages (~6 to 9 years) and includes children’s expanding relationships with caregivers, adults, teachers and peers, spending increasing time in schools, communities and environments beyond home. Cooperation, competition and collaboration are honed, along with motor and academic competencies. Schools and peer activities gain prominence, as social hierarchies become more pronounced and complex, including friendships, bullying and peer pressure.

Although learning and academic skills advance, consequences of early nutritional deficiencies and health risks are expressed during middle childhood. For example, early stunting is associated with poor academic performance and obesity with precursors of type 2 diabetes.^18^ Variation in earlier attachment styles and self-regulation become evident in self-concept, identity and peer relationships (eg, social skills, prosocial behaviours, and regulation of physical and relational aggression). Classroom behavioural interventions can foster self-regulation and prosocial behaviours and decrease behavioural problems that interfere with learning and relationships. High-quality schools can maintain and enhance positive effects of earlier interventions. A recent US national study showed that preprimary educational access and increases in educational spending increased educational attainment and earnings and reduced incarceration.^34^

Most countries provide universal primary schooling; 91% of children aged 6–11 years are enrolled in school globally.^35^ However, inequitable access to quality schooling compromises millions of children’s human capital development.^35^ ‘Learning poverty’ (eg, ‘inability to read and understand simple text by age 10’) compromises children’s academic and earning potential.^36^ Schools’ effectiveness in supporting learning may be hindered by large class sizes and inadequate accountability or support for quality instruction. Interventions that address these learning barriers, including some at scale, have enhanced human capital.^37^

Transforming schools into nurturing institutions that foster learning, health and well-being is central to children thriving. Evidence-based interventions include school meal and health services, such as vision screening, oral health, malaria prevention, human papilloma virus and tetanus toxoid vaccinations.^35 38^ Increasing community responsibility for protection from violence (including bullying and cyberbullying) merges child protection, safety and security with the health, learning and nutritional agendas that schools can integrate so that children thrive.

### Adolescence

Adolescence, a period of transitions, includes puberty, associated growth and hormonal processes, neural reorganisation and expanded cognitive capacities. The WHO, the Adolescents and Youth Constituency of the Partnership for Maternal, Newborn & Child Health, and the UN Major Group for Children and Youth define adolescent well-being as ‘the support, confidence, and resources to thrive in contexts of secure and healthy relationships’.^39^ The domains that support adolescent well-being align well with the NCF, illustrating that adolescence represents the continuation of developmental processes that originate in infancy and continue throughout childhood. The ability to formulate and implement goals, shape responsive relationships and assume responsibility for self and others marks the transition from beneficiary to benefactor of NC in human capital development.

Adolescents have heightened sensitivity to rewards and increased self-awareness, sexual and romantic interests, along with autonomy, agency and social responsibility.^40^ Although increases in sensation-seeking present opportunities for creativity, engagement and personal development, they may also include risk-taking with serious, even life-threatening, consequences (eg, HIV, suicide, violence and substance abuse).^41^ Obesity prevalence increases during adolescence, as do mental health problems.^42^ Adolescent pregnancy and parenthood introduce risks for both generations due to neurophysiological factors, interrupted schooling and other social consequences.^43^

Access to quality education is fundamental to human capital development during adolescence. However, 16% of children aged 12–14 years and 36% of children aged 15–17 years are out of school, often due to family issues, behaviour problems or school unavailability. In 47% of low-income countries, secondary school is neither free nor compulsory.^35^

Families, youth community programmes, schools and workplaces offer opportunities for adolescents to build human capital and compensate for earlier adversities. Families remain important sources of social connectedness. Programmes that focus on parent–youth interactions are effective at reducing behavioural problems and improving life skills among high-risk children and adolescents, illustrating the continued importance of family nurturance.^44^ Youth community programmes support not only academic advances but also transitions to employment, civic engagement, affiliation and leadership, as well as responsible family, peer and romantic relationships.^18^

School-based strategies promote human capital development through providing school-based health services (eg, screening and emergency care); increasing school enrolment and attendance (eg, conditional cash transfers); improving performance (eg, merit-based student scholarships); teaching at appropriate learning levels; decreasing pupil–teacher ratios; implementing youth community programmes; and building new schools.^45^ Schools can also support healthy behaviours and relationships through wellness programmes, nutritious meals, safe spaces and physical activities that can minimise obesity, risky sex and substance use.^18^

Workplaces can encourage training and learning opportunities, healthy relationships with non-parental/family adults and mentoring opportunities, and can address health risks and sexual harassment. Successful programmes have gone beyond classroom-based technical training to provide a range of life skills and vocational and employment services.^46^ Ensuring effective development and implementation of laws and regulations pertaining to child labour, child marriage, treatment of children and youth in justice systems, prohibiting discrimination, and workplace safety can support positive trajectories into adulthood.

## Policies to support NC

Policies and the laws and regulations for their implementation can be effective if they are evidence-based and administered to ensure children’s rights, and provide needed guidance, resources and protection. As illustrated through examples in table 1, across the life course, protective laws and regulations reduce exposure to toxins, increase safety, raise public awareness, require product labelling and ensure healthcare provider access, along with policies that promote parental mental health and protection from violence exposure. Policies, laws and regulations also focus on issues relevant to specific age groups, such as parental leave, breastfeeding policies, universal schooling and child marriage regulations. Although Target 4.2 of the SDGs advocates that girls and boys receive at least 1 year of preprimary education, in some low-income countries, enrolments are below 22%. Policy implementation requires multilevel coordination and careful evaluation of benefits relative to costs to determine successful implementation strategies.^47^

**Table 1:** Examples of policies to support nurturing care from preconception through adolescence

	Preconception/prenatal	Newborn	Infancy	Preschool	Middle childhood	Adolescence
Enabling environments	Universal healthcare, ban on environmental toxins (eg, lead); free preschooling, primary and secondary schooling, child protection; safe water and sanitation; inclusive policies and services for people with disabilities					
Health	Inclusive preventive and promotive quality healthcare for children and adolescents					
	Prenatal services, smoking, drug use cessation	Baby-friendly hospital initiative	Immunisation	Well-child evaluations	Promotion of physical activity	Access to sexual and reproductive services
Nutrition	Equitable access to safe, nutritious and affordable foods					
	Healthy diet, micronutrients, food assistance	Exclusive breastfeeding	Exclusive breastfeeding, complementary feeding, micronutrients	Food assistance, healthy school meals	Food assistance, healthy school meals	Food assistance, healthy school meals
Security and safety	Protection for children and adolescents					
	Violence prevention	Clean water, air, and sanitation	Clean water, air, and sanitation	Injury prevention	Bullying prevention	Prevention of child marriage
Learning	Free and inclusive education					
	Prenatal care	Pregnancy and birth preparation	Parental education and quality care	Quality care, education and parental support	Quality instruction and parental engagement	Vocational and life skills training
Responsive relationships	Care for children and adolescents					
	Prevent gender-based violence	Promote skin-to-skin contact	Parent–infant attachment support	Prevent harsh punishment	Promote prosocial peer relations	Partnership and leadership opportunities

## Strategies to support children and adolescents with additional needs

To pursue equity in child health and development, policies, programmes and services vary in intensity and coverage across target groups. At a population level, implementation of some policies applies universally through services such as free schooling, safe water and violence prevention. For children and adolescents facing specific adversities (eg, disabilites, displacement, conflict, poverty and disasters), universal policies may be inadequate to mitigate multiple risks, and targeted programmes, such as cash transfers and individualised services, may be necessary. For the one-in-six children aged 5–19 years estimated to experience severe disabilities, intensive services are necessary.^48^ Improving health and healthcare for children with additional needs requires integrated services. The core SDG principle to promote equity is especially salient as the NCF is expanded throughout childhood and adolescence.

## Conclusion

The NCF recommends implementation through multisectoral policies, services and programmes that capitalise on the interdependencies across NC components from preconception through adolescence. Simultaneous investments in health, education and social protection, together with bidirectional relations across sectors, can ensure that children are healthy and well nourished, and prepared to learn in the context of supportive home environments and quality educational services.^49^ Multisectoral approaches are particularly important for responding to children’s and adolescents’ needs in low-resource and vulnerable (eg, conflict-affected) settings. Responsive caring relationships reinforce health, nutrition and learning, and help to activate and maintain protection of children from threats.^49^ Multisectoral collaboration can foster positive dynamic developmental trajectories with capabilities to mitigate adversities when necessary and to facilitate an intergenerational progression that builds human capital in future generations.^1 2 49^

Adopting an extended version of the NCF as a comprehensive, evidence-based framework is a critical step in promoting equity and advancing goals that reduce threats of poor health, food insecurity, illiteracy, neglect and cruelty, inadequate resources and limited social freedom. The pathway to achieving these goals is through continua of enablers, support and specialised services for children and adolescents that begin with the family and extend through communities and services to the establishment and implementation of supportive policies and laws.

Attaining the SDGs hinges on children acquiring human capital through developmental trajectories that build their capital, not only early in development but also throughout the first two decades. As global challenges continue and expand, success depends on implementing a comprehensive framework that is broadly focused on the essential components of NC (health, nutrition, learning, responsive relationships, and safety and security), equity and human rights. This over-riding goal is operationalised by the Global Strategy for Women’s, Children’s, and Adolescents’ Health, which provides an action plan to ensure that children survive and thrive in transformed environments that promote their health and well-being.^11^ It is also echoed in the vision that the WHO and UNICEF are taking forward with governments and stakeholders to strengthen policies, systems and services to enable children and adolescents to survive and thrive.^50^

The NCF, embedded in the daily activities of families and sustained by relationships with communities, services and policies, acknowledges the impact of economic, sociopolitical and climatic issues, including unanticipated events, such as pandemics and other humanitarian disasters. With dynamic capacities to mitigate adversities and a philosophical grounding in improving the well-being of the most marginalised groups, the concepts promoted in the NCF, extending from preconception through adolescence, are strategically positioned to enhance human capital, to attain the SDGs and to ensure that all children and adolescents reach their developmental potential.

## Data Availability

There are no data in this work.
